# Supplementary material for: Transitions in sexual behaviour among gay, bisexual, and other men who have sex with men in England: Data from a prospective study
Source: PLoS One. 2025 Mar 4;20(3):e0308238. doi: 10.1371/journal.pone.0308238 (PMC11878903; doi:10.1371/journal.pone.0308238)
Supplement: S1 File — (DOCX) [file pone.0308238.s001.docx]

**Longitudinal changes in condomless anal sex with multiple partners**

**Proportion transitioning or remaining in CLS2+ behaviour**

Longitudinal changes in CLS2+ were graphically visualized and descriptive analysis was done to present changes in men who reported CLS2+ and did not report on this behaviour among those men who reported CLS with two or more partners in the previous visit, and the proportion who reported on this behaviour and did not report CLS with two or more partners among those who did not report CLS with two or more partners in the previous visit. To assess patterns of change, only responses from men who completed at least two consecutive online questionnaires were included.

**Figure 1** shows the change in the proportion reporting CLS with two or more partners, among 622 men, from the first online questionnaire to the last online questionnaire. In this analysis, only responses of behaviour changes (CLS2+ 🡪 CLS2+, CLS2+ 🡪 no CLS2+, and vice versa) were included, while the responses that included missing (missing 🡪 CLS2+, missing 🡪 no CLS2+ and vice versa) were excluded.

Orange colour indicates CLS2+ in the current period, while blue indicates no CLS2+ in the current period. Darker shading (after the first period) indicates that CLS2+ was reported in the previous period. The observed proportions of CLS2+ among the 622 men in the online cohort remained relatively stable: 39.1% at the first online questionnaire (darkest orange) and 40.3% at the ninth online questionnaire (accumulation of medium orange and pale orange). The overall prevalence of CLS with two or more partners among these men across nine questionnaires was 37.9%, similar to those among the 1,162 men. The proportion of men who switched to report CLS with two or more partners, given that they did not report this behaviour at the previous questionnaire, was between 5% and 12% of total respondents (shown in pale orange). While the proportion of men who transitioned out of the ‘higher-risk’ at each questionnaire was between 6% and 14% (shown in medium green).

**Figure 1. Proportion transitioning or remaining in the same frequency of condomless anal sex with two or more partners category among 622 GBMSM in the AURAH2 study during follow-up**


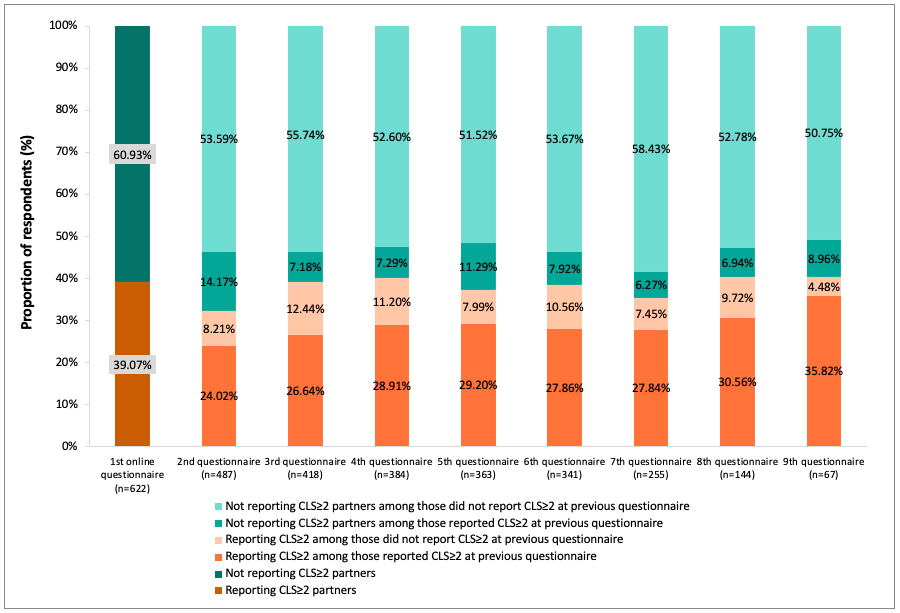


Table 1 illustrates the proportion of changes from a different initial state (higher-risk; lower-risk; skipping questionnaire; loss to follow-up), according to the previous questionnaire among these men, as shown in the sorted lasagna plot. In producing this table, we excluded missing responses due to loss to follow-up (changes from ‘lost’ to ‘lost’).

**Table 2. Individual changes in reporting sexual behaviours among 622 GBMSM in the AURAH2 study over time (based on lasagna plot, excluding questionnaire from lost participants)**


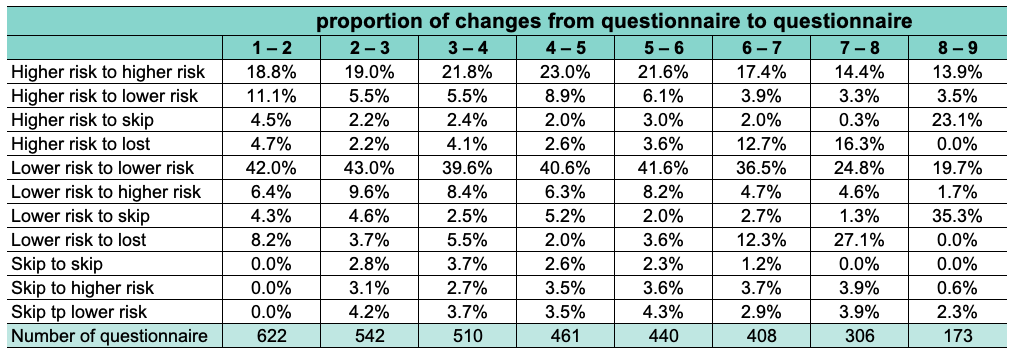


There was high stability among the group of men who reported lower-risk behaviour from one questionnaire to the next, up to the 7^th^ online questionnaire (ranged between 36.5% and 42.0%of all the paired observations, up to the 7^th^ online questionnaire). Men who reported higher-risk behaviour also tended to stay in this behaviour over time until the 9^th^ questionnaire (ranged between 13.9% and 23.0%). The proportions of men who reported an ‘unstable’ trajectory (a switch from lower-risk to higher-risk and vice versa from questionnaire to questionnaire) were relatively small (higher-risk to lower-risk ranged between 3.3% and 11.1%; lower-risk to higher-risk ranged between 1.7% and 9.6%) and tended to decrease over time.
